# A statement of the rights of scientists and engineers

**DOI:** 10.2349/biij.5.2.e10

**Published:** 2009-04-01

**Authors:** W Hendee

**Affiliations:** Editor, Medical Physics, Chair, Publications Committee, International Organization of Medical Physics

## Abstract

As the Editors of the Biomedical Imaging and Intervention Journal, we are pleased to introduce “The Bill of Rights” written by Dr William Hendee, Chair of the Publication Committee of the International Organization of Medical Physics (IOMP). This document covers the fundamental rights and responsibilities of a scientist - not just medical physicists but the entire biomedical imaging community, including the clinicians and researchers. The simultaneous publication of this document in worldwide leading medical physics and allied journals aims to disseminate these standards to the whole scientific world. We, as part of the wider biomedical imaging science community and as the editors of the biij, are fully committed to ensuring these rights are not infringed by anyone, anywhere.

BJJ Abdullah and KH Ng

Editors, Biomedical Imaging and Intervention Journal

## BILL OF RIGHTS FOR SCIENTISTS AND ENGINEERS

### Preamble

A scientist or engineer (S/E) uses understanding, insight and ingenuity to discover new knowledge and to create new technologies that benefit individuals and societies. In pursuing these goals, a S/E must be free to theorize and experiment unimpeded by political pressures, religious dogma or fear of reprisal. Preservation of this freedom is the purpose of the Bill of Rights for Scientists and Engineers. The Bill of Rights is consistent with the Statement on the Universality of Science of the International Council for Science (ICSU).

### Articles

1. A S/E is an individual who uses a scientific approach in the pursuit of new knowledge and technologies. A S/E is not required to possess any specific credential such as an academic degree, appointment in an institution, funding by an agency, or membership in an organization.
2. Science and engineering may be practiced in any location; they are not confined to academic institutions, government facilities, or industrial settings. An individual using a scientific approach in a home laboratory is pursuing science or engineering just as is a S/E employed by an institution, industry or government agency. No prejudice towards the work of a S/E shall be exercised based on an individual’s affiliation or lack thereof with a particular institution, organization or agency.
3. A S/E shall not be dissuaded from pursuing scientific inquiry because of political or religious concerns, or because the inquiry deviates from a conventional perspective.
4. A S/E shall be able to use any approach to new knowledge and technologies, limited only by the restrictions that the approach follows sound scientific principles and does not violate societal ethical precepts.
5. A S/E shall be free to collaborate with other individuals in the same or other locations, with the understanding that collaboration may require covenants protecting confidentiality and intellectual property.
6. A S/E shall not be subject to restraints in the presentation and publication of results that are imposed by political or religious entities or because the findings conflict with traditional knowledge. Scientific and engineering results should always be evaluated on their merits and not because of preconceived notions of “truth”.
7. A S/E shall decide who should coauthor scientific publications based on well-established guidelines for co-authorship. Courtesy authorship to senior personnel in a S/E’s laboratory or institution is unacceptable.
8. A S/E shall strive to ensure that scientific results are widely accessible to the scientific community.
9. A S/E should object to misuse of research findings for political, ideological or financial purposes.
10. At all times a S/E shall adhere to universal ethical and moral standards.

